# Epidemiologic Behavior and Estimation of an Optimal Cut-Off Point for Homeostasis Model Assessment-2 Insulin Resistance: A Report from a Venezuelan Population

**DOI:** 10.1155/2014/616271

**Published:** 2014-10-29

**Authors:** Valmore Bermúdez, Joselyn Rojas, María Sofía Martínez, Vanessa Apruzzese, Mervin Chávez-Castillo, Robys Gonzalez, Yaquelín Torres, Juan Salazar, Luis Bello, Roberto Añez, Maricarmen Chacín, Alexandra Toledo, Mayela Cabrera, Edgardo Mengual, Raquel Ávila, Freddy Pachano, José López-Miranda

**Affiliations:** ^1^Endocrine and Metabolic Diseases Research Center, University of Zulia, Maracaibo 4001, Venezuela; ^2^Institute of Clinical Immunology, University of Los Andes, Mérida 5101, Venezuela; ^3^Institute of Biological Investigations, University of Zulia, Maracaibo 4001, Venezuela; ^4^Pharmacology Department, Physiological Sciences Unit, University of Zulia, Maracaibo 4001, Venezuela; ^5^Morphological Sciences Department, University of Zulia, Maracaibo 4001, Venezuela; ^6^Lipid and Atherosclerosis Unit, Department of Medicine, IMIBIC/Reina Sofia University Hospital/University of Cordoba and CIBER Fisiopatología Obesidad y Nutrición (CIBEROBN), Institute of Health Carlos III, Córdova 14004, Spain

## Abstract

*Background.* Mathematical models such as Homeostasis Model Assessment have gained popularity in the evaluation of insulin resistance (IR). The purpose of this study was to estimate the optimal cut-off point for Homeostasis Model Assessment-2 Insulin Resistance (HOMA2-IR) in an adult population of Maracaibo, Venezuela. * Methods.* Descriptive, cross-sectional study with randomized, multistaged sampling included 2,026 adult individuals. IR was evaluated through HOMA2-IR calculation in 602 metabolically healthy individuals. For cut-off point estimation, two approaches were applied: HOMA2-IR percentile distribution and construction of ROC curves using sensitivity and specificity for selection. * Results.* HOMA2-IR arithmetic mean for the general population was 2.21 ± 1.42, with 2.18 ± 1.37 for women and 2.23 ± 1.47 for men (*P* = 0.466). When calculating HOMA2-IR for the healthy reference population, the resulting p75 was 2.00. Using ROC curves, the selected cut-off point was 1.95, with an area under the curve of 0.801, sensibility of 75.3%, and specificity of 72.8%. * Conclusions.* We propose an optimal cut-off point of 2.00 for HOMA2-IR, offering high sensitivity and specificity, sufficient for proper assessment of IR in the adult population of our city, Maracaibo. The determination of population-specific cut-off points is needed to evaluate risk for public health problems, such as obesity and metabolic syndrome.

## 1. Introduction 

Insulin resistance (IR) is currently one of the most important metabolic risk factors associated with cardiovascular disease [1], type 2 diabetes mellitus (T2DM) [2], and some phenotypes of metabolic syndrome (MS) [3]. As of today, several factors have been related to the progressive loss of tissue-targeted insulin effects including lifestyle behavior [4], environmental factors [5], prenatal reprogramming [6], nutritional patterns [7], physical activity [8], and ethnicity [9]. In spite of its importance during pathogenesis and amplification of disease, there is still controversy regarding which IR evaluation method to apply.

The gold standard for IR estimation is the hyperinsulinemic-euglycemic clamp technique proposed by DeFronzo et al. [10], albeit several limitations when applied to larger populations, such as technical difficulties and high cost, rendered it unviable. Therefore, mathematical models have been devised to measure IR in manner comparable to the hyperglycemic-euglycemic clamp. One of such models is the Homeostasis Model Assessment, first published by Matthews et al. [11] in 1985, which proposed the original HOMA equation (fasting glucose × fasting insulin/22.5), and a recalibrated formula by 1998 [12] labeling it HOMA2-IR. This new model offered several advantages including the calculation of % sensitivity and % beta-cell, using not only fasting levels of glycemia and insulin but also peptide C. In 2004, the University of Oxford launched the HOMA2 Calculator, free software which renders a more precise and fast calculation of HOMA2-IR, determining insulin sensibility and beta-cell function within a range of 1–300 *μ*UI/L for insulin and 20–460 mg/dL for glucose, adjusting this model for hyperinsulinemic or hyperglycemic conditions, hepatic and peripheral IR, and circulating proinsulin [11, 13, 14].

Although the HOMA model has been extensively used, a worldwide cut-off point has not been established, albeit its prerequisite has been strained by the mandatory requirement of population and ethnic specific cut-off points for metabolic indicators, HOMA-IR included [15]. Therefore, 2 approaches have been proposed to determine HOMA-IR cut-off points. The first one uses a certain percentile such as the 95th [16] or the 75th [17] as recommended by Reaven in The First Annual World Congress on the Insulin Resistance Syndrome [18]. The second approach relies on construction of ROC curves derived from a specific population in order to select a valid cut-off point according to sensitivity, specificity, and other indexes [19].

Given the importance of IR quantification during the evaluation of MS components in large populations studies and its requirement in obesity-burdened [20], physically inactive [21], and inflammation-prone [22] communities such as ours, the purpose of this study is to determine the appropriate cut-off point for HOMA2-IR in a representative population sample from the city of Maracaibo, Venezuela.

## 2. Materials and Methods

### 2.1. Population and Sample Selection

The sampling method was already published in the Maracaibo City Metabolic Syndrome Prevalence Study cross-sectional proposal [23]. Briefly, population estimations for Maracaibo city (the second largest city of Venezuela) from the National Institute of Statistics were used (1,428,043 by 2007), the sample was calculated to be 1,986 individuals and the overall number of individuals was 2,230. A total of 244 subjects (12%) were added for oversampling [23], in order to increase accuracy of the estimates obtained from smaller subgroups from the overall sample [23, 24]. The city of Maracaibo is divided into parishes and each of these was proportionally sampled in a multistage cluster method; during the first stage, the cluster was represented by sectors from each of the 18 parishes, finally selecting 4 from each parish by simple random sample. In the second phase, the clusters were represented by city blocks within the sectors, in which they were selected by simple random sample using a random number generation tool. From an overall population, 2,026 individuals were selected on the basis of availability of insulin determination.

For the determination of the HOMA2-IR cut-off point, a reference population of 602 healthy individuals was selected based on the exclusion of subjects with the following conditions: obesity, MS, hypertension, type 1 diabetes mellitus, thyroid or hepatic disease, coronary artery disease, heart rhythm disorders or cerebrovascular disease, polycystic ovary syndrome, and consumption of medication which may influence fasting blood glucose or lipid profile. All the individuals enrolled in the study signed a written consent before physical examination and anamnesis and all procedures were approved by the Ethics Committee of the Endocrine and Metabolic Diseases Research Center of The University of Zulia, Maracaibo, Venezuela.

### 2.2. Clinical Evaluation

The assessment of blood pressure was done using a calibrated mercury sphygmomanometer, with the patient previously rested (for a minimum of 15 minutes) in a sitting position with both feet touching the floor. The arm was positioned at heart level, and a properly sized cuff was used for the procedure. Systolic blood pressure was determined at the first Korotkoff sound, whereas diastolic blood pressure was determined at the fifth Korotkoff sound. Blood pressure values were determined twice, with an interval of at least 15 minutes, and the results were averaged. Blood pressure classification was completed using the criteria proposed in the VII Joint National Committee (JNC-7) [25]. Weight was assessed using a digital scale (Tanita, TBF-310 GS Body Composition Analyzer, Tokyo, Japan), while height was obtained with a calibrated rod; the subjects were barefooted and wearing light clothing at all times. Body mass index formula was applied to all individuals (Weight/Height^2^) and categorization was done using the WHO classification [26]. Waist circumference (WC) was measured using calibrated measuring tape in accordance with the anatomical landmarks proposed by the USA National Institutes of Health protocol [27]. MS was diagnosed using the criteria from the IDF/AHA/NHLBI-2009 consensus [28].

### 2.3. Laboratory Workup

Overnight fasting determination of glucose, total cholesterol, triglycerides, and HDL-C was done with an automated analyzer (Human Gesellschaft für Biochemica und Diagnostica mbH, Germany); the intra-assay variation coefficient for the total cholesterol, TAG, and HDL-C was 3%, 5%, and 5%, respectively. LDL-C and VLDL-C levels were calculated applying the Friedewald formula [29] only if triglycerides were below 400 mg/dL; if they were above the mentioned cut-off point, LDL-C concentrations were measured through lipoprotein electrophoresis and densitometry with a BioRad GS-800 (BioRad). Insulin was determined using an ultrasensitive ELISA double-sandwich method (DRG Instruments GmbH, Germany, Inc.).

### 2.4. Statistical Analysis

Qualitative variables were expressed as absolute and relative frequencies, considering the results statistically significant when *P* < 0.05 in either the* Z* test for proportions or the *χ* ^2^ test when applied. No normally distributed quantitative variables were subjected to logarithmic transformation observing a normal distribution after Geary test; results were expressed as mean ± standard deviation. To determine differences between means, Student's* t*-test was applied (when comparing two groups) or one-way ANOVA (when comparing three or more groups) complemented with the post hoc Tukey test. Data were analyzed using the Statistical Software for Social Sciences (SPSS version 20 for Windows, Chicago, IL, USA).

**Figure 1:**
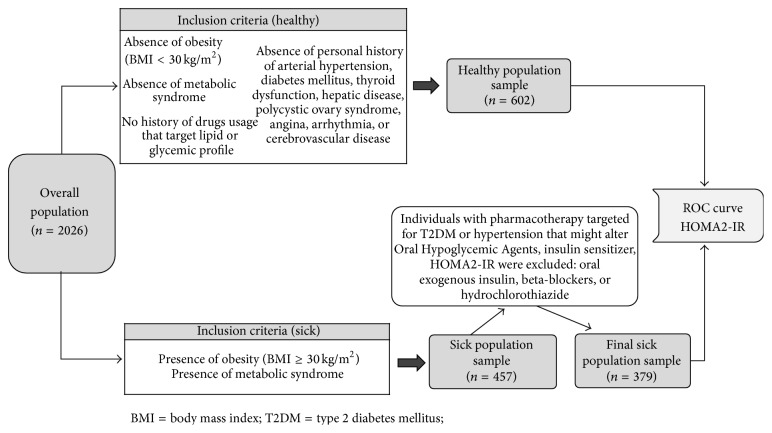
Methodology for selecting healthy and sick subject groups in order to construct ROC curves used for determination of HOMA2-IR cut-offs.

HOMA2-IR was calculated using the software supplied by the Oxford Centre for Diabetes Endocrinology and Metabolism available at http://www.dtu.ox.ac.uk/homacalculator/index.php. In order to determine a proper cut-off point, a reference population was selected, of which the primary results were distributed in percentiles (5-25-50-75-95), with the 75th percentile chosen as the cut-off for HOMA2-IR based on recommendations by Reaven [18]. To further ascertain the HOMA2-IR cut-off, a receiving operating characteristic curve was constructed based on the aforementioned reference population and a selected metabolically unhealthy population of 379 subjects. This sick population was comprised of subjects complying with either or both of the following inclusion criteria: presence of obesity and presence of MS [30]. These criteria yielded a preliminary group of 457 subjects, which was reduced to final sick sample of 379 after exclusion of subjects currently consuming medication which may influence glycemic or lipid profiles (such as hypoglycemic agents, insulin sensitizers, insulin, beta-blockers, or hydrochlorothiazide), as shown in Figure 1. Three separate ROC curves were constructed, one for females, one for males, and one for merging both genders. The comparison between AUC by sex was assessed using DeLong's test [31]. To establish the optimal cut-off for HOMA2-IR, the following indexes were used [32]: Youden's index, the distance closest to ROC (0.1), and positive likelihood ratio.

## 3. Results

### 3.1. General Characteristics of the Selected Sample

A total of 2,026 subjects were studied, 52.1% of whom were female (*n* = 1056) and 47.9% were male (*n* = 970). The mean age was 49.7 ± 15.4 years. Overall arithmetic mean for HOMA2-IR was 2.21 ± 1.42, with 2.18 ± 1.37 and 2.23 ± 1.47 for women and men, respectively; *P* = 0.466. The metabolic and anthropometric characteristics of this sample are shown in Table 1.

### 3.2. Insulin Sensibility by Age Groups

**Figure 2:**
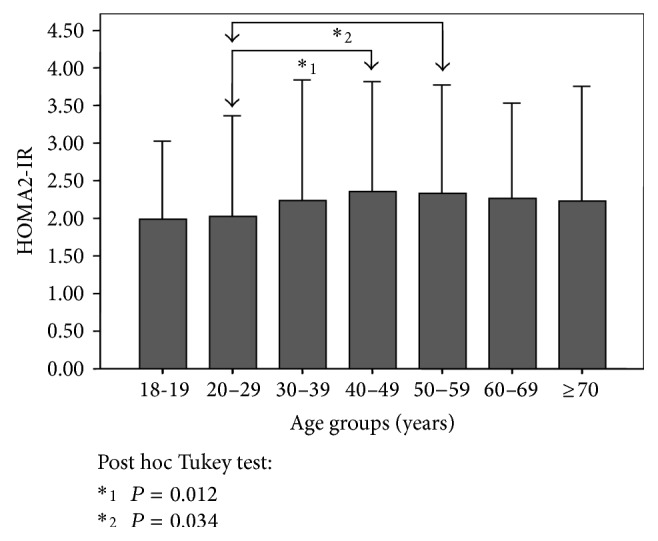
HOMA2-IR according to age groups in adult subjects (*n* = 2026). The Maracaibo City Metabolic Syndrome Prevalence Study, 2014.

**Figure 3:**
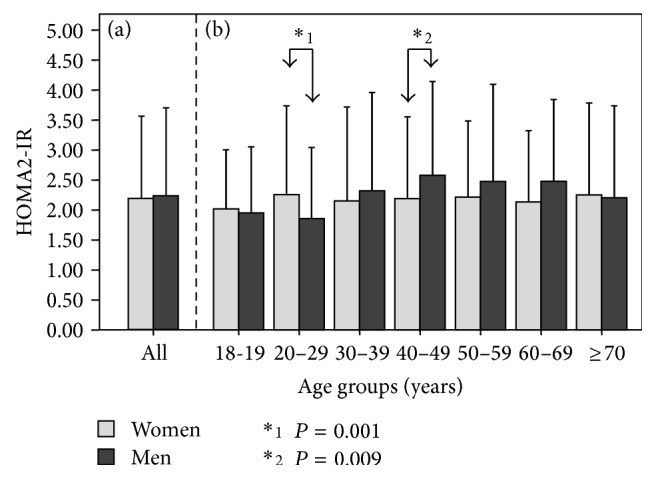
HOMA2-IR according to age groups and gender (*n* = 2026). The Maracaibo City Metabolic Syndrome Prevalence Study, 2014.

Figure 2 shows HOMA2-IR values according to age groups. Significant differences were observed between groups aged 20–29 and 40–49 years (2.03 ± 1.34 versus 2.35 ± 1.46, resp.; *P* = 0.012), as well as 20–29 and 50–59 years (2.03 ± 1.34 versus 2.34 ± 1.44, resp.; *P* = 0.034). Likewise, Figure 3(a) displays HOMA2-IR means according to gender, whereas Figure 3(b) depicts these values by gender and age groups. Statistical differences between genders were found within the groups aged 20–29 years (women 2.25 ± 1.48 versus men 1.87 ± 1.20; *P* = 0.001) and 40–49 years (women 2.19 ± 1.36 versus men 2.57 ± 1.56; *P* = 0.009).

### 3.3. Insulin Sensitivity and Body Mass Index

**Figure 4:**
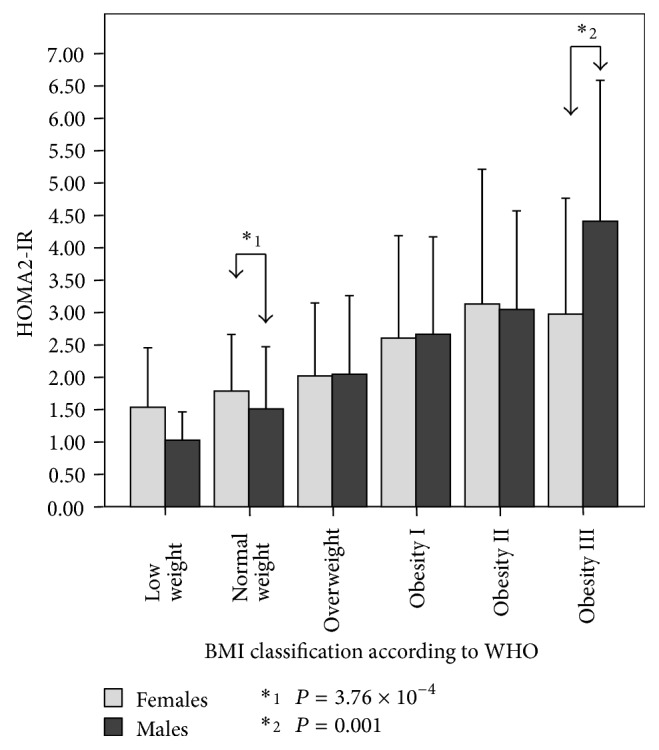
HOMA2-IR according to gender and BMI categories in adult subjects (*n* = 2026). The Maracaibo City Metabolic Syndrome Prevalence Study, 2014.

HOMA2-IR according to BMI classification and sex is observed in Figure 4. A progressive elevation in HOMA2-IR is observed as BMI increases. Indeed, women with low weight had HOMA2-IR values of 1.53 ± 0.92, while obese class III females had 2.97 ± 1.79. Similarly, men with low weight had 1.03 ± 0.44, while the obese class III had 4.41 ± 2.17. These differences regarding BMI were statistically significant for both genders.  Table 2 shows the *P* values for arithmetic mean BMI categories comparisons between men and women. When comparing HOMA2-IR means between females and males, differences were found within the normal weight category, where women obtained the highest results (1.51 ± 0.96 versus 1.79 ± 10.88, resp.; *P* = 3.76 × 10^−4^), and within the obese class III category, where the men had higher values (4.41 ± 2.17 versus 2.97 ± 1.79; *P* = 0.001).

### 3.4. Insulin Sensitivity and Waist Circumference

**Figure 5:**
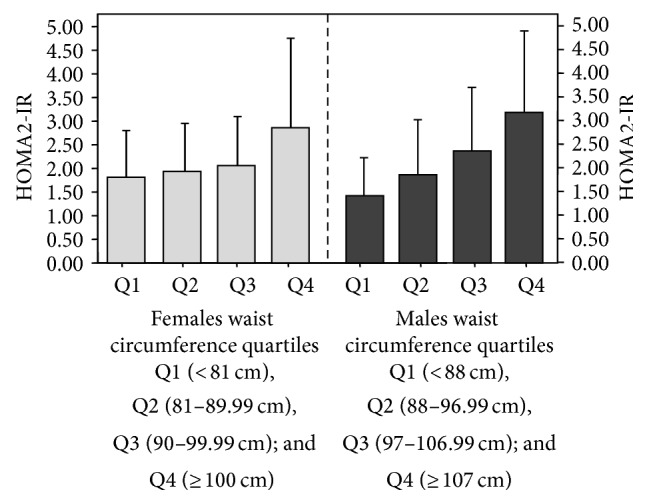
HOMA2-IR according to gender and waist circumference quartiles in adult subjects (*n* = 2026). The Maracaibo City Metabolic Syndrome Prevalence Study, 2014.* Females*: ANOVA: *P* = 6.49 × 10^−22^: post hoc Tukey test: Q1 versus Q4: *P* = 4.00 × 10^−13^, Q2 versus Q4: *P* = 4.05 × 10^−13^, Q3 versus Q4: *P* = 1.35 × 10^−11^.* Males*: ANOVA: *P* = 1.53 × 10^−33^ post hoc Tukey test: *P* < 0.001 for all categories.

Figure 5 exhibits HOMA2-IR according to WC quartiles for men and women, observing a progressive increase along the categories, with 1.81 ± 0.99 in the 1st quartile and 2.86 ± 1.90 in the 4th quartile for women and 1.48 ± 0.88 for the 1st quartile and 3.10 ± 1.56 for 4th quartile. Differences between genders were found within the 1st quartile (*P* = 3.99 × 10^−3^) and 4th quartile (*P* = 0.017).

### 3.5. Reference Population Characteristics and HOMA2-IR Cut-Off Point

The selected reference population (*n* = 602) were constituted by a healthy group of 301 women (48.6%) and 318 men (51.4%).  Table 3 shows the general characteristics of this group. Following recommendations by Reaven [18]—who proposed the p75th for determining HOMA2-IR cut-offs—a preliminary value of 2.00 was selected for both men and women. When assessing by gender, the p75th value for women was 2.10, whereas men showed a p75th of 1.90. Percentile distribution of HOMA2-IR values in reference population is shown in Table 4.

**Figure 6:**
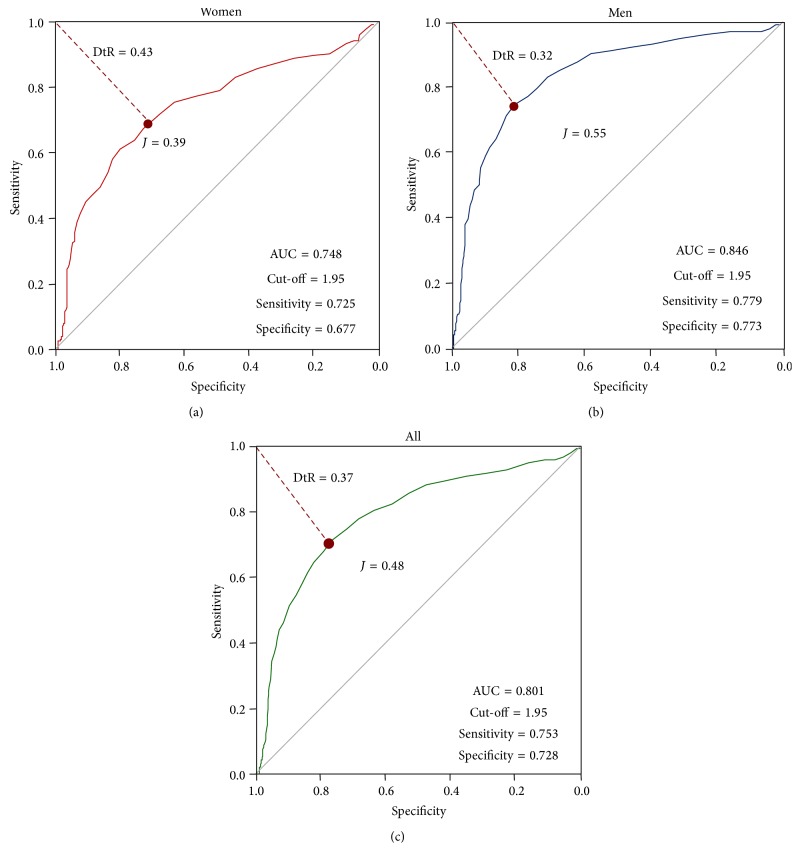
ROC curves constructed to determine HOMA2-IR cut-offs between healthy and diseased subjects. The Maracaibo City Metabolic Syndrome Prevalence Study, 2014.

In order to further explore HOMA2-IR cut-off determination, ROC curves were constructed based on the selection methodology shown in Figure 1. The ROC curve based on both males and females rendered a cut-off value of 1.95 (AUC 0.801), with 75.3% sensitivity and 72.8% specificity.  Figure 6 shows the resulting ROC curve for women, with a cut-off point of 1.95 (AUC 0.748) with 72.5% sensitivity and 67.7% specificity; the ROC curve for men rendered a cut-off point of 1.95 (AUC 0.846) with 77.9% sensitivity and 77.3% specificity. DeLong's test shows nonsignificant differences between the AUC of ROC curves for men and women; *P* = 0.265. Based on the values of sensitivity and specificity, 1.95 was selected as the best HOMA2-IR cut-off value (Table 5).

## 4. Discussion

As previously stated, IR has been associated with several metabolic disorders, including cardiovascular disease [1], T2DM [2], MS [3], metabolic reprogramming during fetal life [6], and physical inactivity [7]. Such role has been fundamental in order to promote knowledge concerning pathogenesis of such diseases and to properly choose potential pharmacological targets to manage them. The current gold standard for the evaluation of insulin sensitivity is the Glucose Clamp Technique [10]. The latest methods are mathematical in concept, and one of them is the HOMA-IR equation proposed by Matthews et al. [11] and its upgraded version, HOMA2-IR, published by Levy et al. [12]. HOMA2-IR has been validated for Latin American populations as seen in the BRAMS project from Brazil [33], a multicentric study which showed both HOMA-IR and HOMA2-IR to be applicable in epidemiological vigilance for MS and IR, with cut-off points of 2.3 for HOMA-IR and 1.4 for HOMA2-IR. Moreover, Garmendia et al. [17] reported a HOMA-IR cut-off of 2.6 for elderly Chilean subjects and Buccini and Wolftbal [34] reported a HOMA-IR cut-off point of 2.64 and finally a 1.67 cut-point for HOMA2-IR in a small Argentinean cohort (*n* = 208).

Despite the importance of IR in the development, progression, and end-organ damage in MS, T2DM, and their comorbidities, there is no consensus regarding optimal cut-off values, particularly in our country. Therefore, the purpose of this investigation was to determine an appropriate cut-off point for HOMA2-IR using ROC curves. This approach in data analysis requires determination of suitable populations to serve as reference or control/healthy individuals, while the remaining individuals were sorted to obtain an appropriate “sick” population. Both of these components are primary materials in the construction of the ROC curve and the selection of cut-offs (19,32). The selection of the cut-off point for HOMA2-IR was performed through two approaches: (a) selection of p75 values, as recommended by Reaven [18], and (b) construction of ROC curves in a reference population. First, according to the percentile distribution of HOMA2-IR from the reference sample (*n* = 602), the resulting p75 was 2.00. Then, after constructing ROC curves, the selected cut-point was 1.95 with corresponding sensitivity of 71.8% and specificity of 77.8%. Interestingly, these approaches rendered similar cut-offs, confirming and supporting one another, suggesting that Reaven was right in recommending the p75 values as reference [18].

When comparing our results to those from Brazil, Chile, and Argentina, our cut-point is 0.3–0.6 points higher, which can be ascribed to sociodemographic and nutritional differences inherent to these populations. In effect, despite a tendency towards growing obesity prevalence currently entailing all of Latin America, obesity figures appear to be higher in our country than in the other aforementioned territories [20], reinforcing the need for local intervals to evaluate insulin sensitivity. Indeed, proper evaluation of cardiometabolic risk factors such as IR through the HOMA2-IR equation is one of the most important tools when assessing epidemiologic risk in a population, particularly in ours, which boasts alarming figures such as 68.1% of elevated BMI (≥25 kg/m^2^) and 42.4% prevalence of MS [20].

The San Antonio Heart Study, one of the largest prospective studies undertaken in the United States, comparing cardiovascular risk factors in Mexican-Americans and non-Hispanic whites, has reported that cardiovascular events increase as HOMA-IR quintiles elevate as well, even after adjustment for age, sex, and ethnic group resulting in an OR of 2.52 (95% CI 1.46–4.36, *P* < 0.0001) [35]. These results are similar to those obtained from the Verona Diabetes Complications Study [36], which published that HOMA-IR is an independent predictor of cardiovascular disease in T2DM subjects, shedding light once more on the imminent need for proper diagnosis and management of insulin resistance in primary and secondary prevention.

At first glance, IR shows an increasing tendency according to age, with the highest peak found at midlife (Figure 2) and predominantly in men over women (Figure 3). Interestingly, within the group aged 20–29 years, women obtained higher IR values than men, related to higher levels of physical inactivity, which coincides with previous findings in our locality [21]. Moreover, IR increases as BMI and WC rise, being higher in women within the normal weight category, whereas it was higher in men within the obesity class III category. These results demonstrate that IR in normal weight subjects is higher for women; and in obese groups, IR is higher for men. This dichotomy could be attributed to visceral adipose tissue quality variance and adipose distribution [37].

As indicated by previous research, the population of Maracaibo has an alarmingly elevated prevalence of obesity, with 33.3% of the sample classified as obese and 34.8% as overweight [20], associated with 59.06% prevalence of physical inactivity [21] and significant low grade inflammation [22]. Insulin resistance states have been associated with oxidized low-density lipoproteins in Latino individuals [38], elevated levels of apoB [39], and higher lipoprotein insulin resistance index suggesting association with lipoprotein particle size and cardiovascular risk [40] and vascular markers of inflammation [41]. Moreover, Vella et al. [42] reported that insulin resistance surrogates, such as HOMA-IR, were associated with cardiovascular disease risk in Hispanic normal weight women; in this regard, our team previously published that as HOMA2-IR increased, so did cardiovascular risk calculated with a correction of the Framingham-Wilson equation, being highest in insulin resistant subjects [43].

Last, yet equally important, is the fact that it has been suggested that Amerindian descendants have higher HOMA-IR indexes [44], which would suggest that all Latino populations would have different IR results due to ethnicity influences, enhancing its role as a nonmodifiable cardiovascular risk factor [9]. The selection of an appropriate population-specific cutoff is of great importance, not only because it enhances accuracy of diagnosis but also because it is adapted to the socioeconomic and genetic factors [20], especially when results are bound to be compared with other countries. As a matter of fact, our cut-off points are different than those found in other Latino countries such as Argentina, a country that also has a very unique genetic admixture [45]. If genetics influences are as important as it would seem to be, then all metabolic variables and anthropometric measurements must be selected according to ethnicity and population [44, 46], validating the need for studies such as this one.

In conclusion, we propose an optimal cut-off value of 2.00 for HOMA2-IR for the evaluation of IR by this mathematical method. This interval offers great sensitivity and specificity, sufficient for proper assessment of IR in the adult population of Maracaibo. Population-specific reference values are required for accurate risk assessment and preventive planning in regard to public health problems such as obesity, T2DM, MS, and cardiovascular disease.

## Figures and Tables

**Table 1:** General characteristics of the population (*n* = 2026). The Maracaibo City Metabolic Syndrome Prevalence Study, 2014.

	Females (*n* = 1056; 52.1%)	Males (*n* = 970; 47.9%)	*P* ^*^
	Mean ± SD	Mean ± SD	
Age (years)	41.0 ± 15.7	38.2 ± 14.9	1.20 × 10^−4^
BMI (kg/m^2^)	27.9 ± 6.2	28.8 ± 6.2	1.00 × 10^−4^
Waist circumference (cm)	91.1 ± 13.7	98.7 ± 15.9	7.64 × 10^−31^
Fasting glycemia (mg/dL)	98.6 ± 31.5	99.6 ± 33.9	0.625
Fasting insulin (*µ*U/mL)	14.5 ± 9.3	14.8 ± 9.8	0.715
HOMA2-IR	2.18 ± 1.37	2.23 ± 1.47	0.466
Total cholesterol (mg/dL)	194.7 ± 44.7	188 ± 47.5	1.97 × 10^−4^
Triacylglycerides (mg/dL)	117.1 ± 85.4	146.2 ± 116.5	1.18 × 10^−13^
VLDL-C (mg/dL)	23.4 ± 17.1	29.2 ± 23.3	2.58 × 10^−10^
LDL-C (mg/dL)	123.8 ± 38.3	118.6 ± 38.7	0.002
HDL-C (mg/dL)	46.9 ± 11.8	40.8 ± 11.3	9.86 × 10^−36^
SBP (mmHg)	117.6 ± 17.4	122.1 ± 15.9	2.65 × 10^−11^
DBP (mmHg)	75.5 ± 10.8	79.1 ± 11.5	3.38 × 10^−13^

^*^Student's *t*-test after logarithmic transformation.

BMI: body mass index; VLDL-C: very low-density lipoprotein cholesterol; LDL-C: low-density lipoprotein cholesterol; HDL-C: high-density lipoprotein cholesterol; DBP: diastolic blood pressure; SBP: systolic blood pressure.

**Table 2:** Results of one-way ANOVA test assessing HOMA2-IR by BMI categories for each gender (*n* = 2026). The Maracaibo City Metabolic Syndrome Prevalence Study, 2014.

	BMI	Low weight	Normal weight	Overweight	Obesity I	Obesity II	Obesity III
Females	Low weight	—	NS	NS	4.86 × 10^−4^	1.42 × 10^−7^	4.67 × 10^−5^
	Normal weight	NS	—	NS	1.85 × 10^−11^	4.47 × 10^−13^	1.05 × 10^−7^
	Overweight	NS	NS	—	6.02 × 10^−6^	1.00 × 10^−11^	4.76 × 10^−5^
	Obesity I	4.86 × 10^−4^	1.85 × 10^−11^	6.02 × 10^−6^	—	0.015	NS
	Obesity II	1.42 × 10^−7^	4.47 × 10^−13^	1.00 × 10^−11^	0.015	—	NS
	Obesity III	4.67 × 10^−5^	1.05 × 10^−7^	4.76 × 10^−5^	NS	NS	—

Males	Low weight	—	NS	NS	0.001	2.39 × 10^−5^	1.24 × 10^−12^
	Normal weight	NS	—	1.23 × 10^−5^	4.40 × 10^−13^	4.40 × 10^−13^	4.40 × 10^−13^
	Overweight	NS	1.23 × 10^−5^	—	5.10 × 10^−7^	5.62 × 10^−9^	4.40 × 10^−13^
	Obesity I	0.001	4.40 × 10^−13^	5.10 × 10^−7^	—	NS	6.10 × 10^−13^
	Obesity II	2.39 × 10^−5^	4.40 × 10^−13^	5.62 × 10^−9^	NS	—	8.43 × 10^−7^
	Obesity III	1.24 × 10^−12^	4.40 × 10^−13^	4.40 × 10^−13^	6.10 × 10^−13^	8.43 × 10^−7^	—

NS: no significance.

**Table 3:** General characteristics of the reference population (*n* = 602) according to gender. The Maracaibo City Metabolic Syndrome Prevalence Study, 2014.

	Women (*n* = 285; 47.3%)		Men (*n* = 317; 52.7%)		*P* ^*^
	Mean	SD	Mean	SD	
Age (years)	29.7	10.9	29.5	11.6	0.768
BMI (kg/m^2^)	23.5	3.2	24.5	3.1	2.8 × 10^−5^
WC (cm)	81.3	8.7	86.8	8.6	2.8 × 10^−14^
Fasting glycemia (mg/dL)	88.4	9.0	87.3	10.6	0.106
Fasting insulin (*µ*U/mL)	12.6	7.8	10.6	6.5	0.001
HOMA2-IR	1.82	1.04	1.55	0.92	0.001
Total cholesterol (mg/dL)	172.0	35.5	175.9	42.3	0.431
Triacylglycerides (mg/dL)	74.4	35.4	95.9	54.3	3.0 × 10^−19^
VLDL-C (mg/dL)	14.88	7.0	19.1	10.8	2.0 × 10^−8^
LDL-C (mg/dL)	107.5	31.6	110.8	37.7	0.847
HDL-C (mg/dL)	49.7	11.9	45.8	12.9	8.2 × 10^−6^
SBP (mmHg)	107.9	9.6	114.3	11.7	4.4 × 10^−12^
DBP (mmHg)	69.9	8.2	73.5	9.1	1.4 × 10^−6^

^*^Student's *t*-test after logarithmic transformation.

BMI: body mass index; VLDL-C: very low-density lipoprotein cholesterol; LDL-C: low-density lipoprotein cholesterol; HDL-C: high-density lipoprotein cholesterol; DBP: diastolic blood pressure; SBP: systolic blood pressure.

**Table 4:** HOMA2-IR percentiles obtained from the reference healthy population (*n* = 602). The Maracaibo City Metabolic Syndrome Prevalence Study, 2014.

	HOMA2-IR				
	p05th	p25th	p50th	p75th	p95th
Gender					
Females	0.80	1.20	1.70	2.10	3.20
Males	0.60	1.00	1.30	1.90	3.20
Age group (year)					
18-19	0.90	1.20	1.70	2.30	3.30
20–29	0.60	1.10	1.40	2.00	3.30
30–39	0.50	1.10	1.50	2.10	3.00
40–49	0.70	1.10	1.40	1.70	2.20
50–59	0.60	1.00	1.40	1.80	2.40
60–69	0.90	1.00	1.30	1.80	2.20
≥70	0.70	0.70	1.30	2.40	2.40
Total	**0.60**	**1.10**	**1.50**	**2.00**	**3.20**

There were no significant differences according to age (one-way ANOVA test, *P* = 0.114).

**Table 5:** HOMA2-IR cut-off points based on ROC curves, sensitivity, specificity, Youden's index, positive likelihood, and distance to the ROC curve. The Maracaibo City Metabolic Syndrome Prevalence Study, 2014.

	HOMA2-IR	Sensitivity (%)	Specificity (%)	Youden's index	Distance to ROC	LR+
Women	2.05	68.0	73.0	0.41^Ψ^	0.41^§^	2.51
	1.95^¶^	72.5	67.7	0.40	0.42	2.24
	1.85	75.8	63.2	0.39	0.44	2.05

Men	2.05	75.3	81.4	0.57^Ψ^	0.30^§^	4.04
	1.95^¶^	77.9	77.3	0.55	0.31	3.43
	1.85	79.6	74.4	0.46	0.32	3.11

All	2.05	71.8	77.4	0.49^Ψ^	0.36^§^	3.17
	**1.95** ^¶^	**75.3**	**72.8**	**0.48**	**0.37**	**2.76**
	1.85	77.8	69.1	0.47	0.38	2.51

^¶^Selected cut-off (HOMA2-IR) based on sensitivity, specificity, Youden's index, and positive likelihood ratios (LR+), giving emphasis to highest sensitivity values.

^Ψ^Cut-off 1 asserted using the maximum Youden's index.

^§^Cut-off 2 obtained from the point closest to ROC (0.1).
